# Integrated bioinformatics and wet-lab analysis revealed cell adhesion prominent genes CDC42, TAGLN and GSN as prognostic biomarkers in colonic-polyp lesions

**DOI:** 10.1038/s41598-023-37501-6

**Published:** 2023-06-26

**Authors:** Elmira Sadat Tabatabaei, Radman Mazloomnejad, Leili Rejali, Flora Forouzesh, Fatemeh Naderi-Noukabadi, Binazir Khanabadi, Zahra Salehi, Ehsan Nazemalhosseini-Mojarad

**Affiliations:** 1 Department of Genetics, Faculty of Advanced Science and Technology, Tehran Medical Science, Islamic Azad University, Tehran, Iran; 2 Basic and Molecular Epidemiology of Gastrointestinal Disorders Research Center, Research Institute for Gastroenterology and Liver Diseases, Shahid Beheshti University of Medical Sciences, P.O. Box 19857-17413, Tehran, Iran; 3 Department of Immunology, School of Medicine, Tehran University of Medical Sciences, Tehran, Iran; 4 Gastroenterology and Liver Diseases Research Center, Research Institute for Gastroenterology and Liver Diseases, Shahid Beheshti University of Medical Sciences, Yeman St, Chamran Expressway, P.O. Box 19857-17413, Tehran, Iran

**Keywords:** Cancer, Computational biology and bioinformatics, Genetics, Molecular biology, Systems biology

## Abstract

Colorectal cancers are derived from intestinal polyps. Normally, alterations in cell adhesion genes expression cause deviation from the normal cell cycle, leading to cancer development, progression, and invasion. The present study aimed to investigate the elusive expression pattern of CDC42, TAGLN, and GSN genes in patients with high and low-risk polyp samples, and also colorectal cancer patients and their adjacent normal tissues. In upcoming study, 40 biopsy samples from Taleghani Hospital (Tehran, Iran) were collected, consisting of 20 colon polyps and 20 paired adjacent normal tissues. The expression of the nominated genes *CDC42*, *TAGLN*, and *GSN* was analyzed using quantitative polymerase chain reaction (Q-PCR) and relative quantification was determined using the 2^−ΔΔCt^ method. ROC curve analysis was performed to compare high-risk and low-risk polyps for the investigated genes. The expression of adhesion molecule genes was also evaluated using TCGA data and the correlation between adhesion molecule gene expression and immunophenotype was analyzed. The role of mi-RNAs and lncRNAs in overexpression of adhesion molecule genes was studied. Lastly, GO and KEGG were performed to identify pathways related to adhesion molecule genes expression in healthy, normal adjacent, and COAD tissues. The results showed that the expression patterns of these genes were significantly elevated in high-risk adenomas compared to low-risk polyps and normal tissues and were associated with various clinicopathological characteristics. The estimated AUC for CDC42, TAGLN, and GSN were 0.87, 0.77, and 0.80, respectively. The study also analyzed COAD cancer patient data and found that the selected gene expression in cancer patients was significantly reduced compared to high-risk polyps and healthy tissues. Survival analysis showed that while the expression level of the GSN gene had no significant relationship with survival rate, the expression of CDC42 and TAGLN genes did have a meaningful relationship, but with opposite effects, suggesting the potential use of these genes as diagnostic or prognostic markers for colorectal cancer. The present study's findings suggest that the expression pattern of CDC42, TAGLN, and GSN genes was significantly increased during the transformation of normal tissue to polyp lesions, indicating their potential as prognostic biomarkers for colorectal polyp development. Further results provide valuable insights into the potential use of these genes as diagnostic or prognostic markers for colorectal cancer. However, further studies are necessary to validate these findings in larger cohorts and to explore the underlying mechanisms of these genes in the development and progression of colorectal cancer.

## Introduction

Colorectal cancer is the third most widely diagnosed cancer and the second major cause of cancer death^1^. Most of colorectal cancers (> 90%) are malignant neoplasm, which grows from glandular epithelial cells of the colon and rectum, called colorectal adenocarcinoma (COAD)^2^. The development of COAD can be divided into four stages: (1) Initiation, which involves irreversible genetic damage that predisposes affected cells to neoplasia; (2) Promotion, characterized by the induction of abnormal growth; (3) Progression, resulting from genetic and epigenetic alterations in tumor cells; and (4) Metastasis, marked by the spread of cancer cells throughout the body^3^. Colorectal polyps can be a precursor of colorectal cancer and their early detection and removal can prevent the development of the malignant disease. There are several types of colorectal polyps including adenomatous polyps (tubular, villous, and tubulovillous), inflammatory polyps, hamartomatous polyps, and hyperplastic polyps^4^. Among these, adenomatous polyps are considered the most likely to develop into colorectal cancer, while other types such as hyperplastic and inflammatory polyps have lower risk^5^. The main medical classification of polyps is based on the distinction between high-risk and low-risk polyps, as determined by clinical parameters. High risk adenomas (HRA) include (a) a tubular adenoma ≥ 1 cm in size, (b) three or more adenomas, or (c) an adenoma with villous histology or high-grade dysplasia^4^. Conversely, low-risk adenomas (LRA) are defined as (i) 1 to 2 tubular adenomas < 1 cm in size, (ii) without a villous component, or (iii) without high-grade dysplasia^6^.

In the development of cancer and precancerous tissues, various cellular and molecular functions are involved, including defects in DNA repair, gene transcription, cell cycle control, cell adhesion, and cell death^7–11^. Among them, cell adhesion is the ability of a single cell to attach to another cell or extracellular matrix (ECM). Cell adhesion plays a vital role in the communication and regulation of cellular processes and is essential in developing and maintaining tissues. Cell adhesion is involved in the stimulation signals that regulates cell differentiation, cell cycle, cell migration, and cell survival^6^. Cellular adhesion molecules can be divided into four main groups based on their protein sequence and structure: cadherins, integrins, selectins, and immunoglobulins. This division is strongly associated with distinct types of cell junctions made by classes of proteins expressed at the cell surface^12^.

In the context of colorectal cancer, the genes CDC42, TAGLN, and GSN have gained increasing recognition due to their significant involvement in key processes related to cancer progression and metastasis. Aberrant expression of these genes has been consistently reported in CRC, and their dysregulation is associated with enhanced cell migration, invasion, and poor prognosis in patients^21^.

CDC42, a member of the Rho family of small GTPases, plays a crucial role in regulating various cellular processes, including cell-to-cell adhesion, cell proliferation, and the cell cycle. Its involvement in the regulation of the actin cytoskeleton allows CDC42 to control both cell architecture and motility^12^. Cancer metastasis heavily relies on the migration of cancer cells and their ability to invade the surrounding tissue, breach the basement membrane, and enter the circulation. Single-cell migration is believed to involve a process known as epithelial-to-mesenchymal transition (EMT), where tumor cells dissolve their cell-to-cell contacts and acquire a more mesenchymal morphology, facilitating their movement through the extracellular matrix^13^. Notably, activation of CDC42 during mesenchymal cell movement is essential. Previous studies indicate an important role for CDC42 in EMT-mediated migration and invasion of individual tumor cells^14^. Overexpression of CDC42 has also been associated with several other cancers^15^.

TAGLN, a member of the calponin family, exhibits abnormal expression in various diseases, particularly cancers^16^. It is implicated in stress fiber formation, playing a central role in cell adhesion and morphogenesis^17^. TAGLN is predominantly expressed in fibroblasts and smooth muscle cells. Additionally, TAGLN is involved in the disintegration of the extracellular matrix (ECM) and angiogenesis during smooth muscle development, stem cell differentiation, and embryonic blood vessel formation, thereby contributing to tumor cell invasion and angiogenesis^13^. TAGLN, also known as SM22, is a TGFβ-inducible gene highly expressed in numerous tissues, such as the prostate, bladder, stomach, colon, and uterus. TAGLN2, an oncogene, regulates various biological processes in cancer cells, including proliferation, differentiation, and apoptosis^18^.

GSN is another adhesion-related gene that is critical in regulating cellular functions. It is a multifunctional protein that reshapes cytoskeleton structures and is crucial in controlling cellular interactions^19^. During EMT, the loss of E-cadherin expression is a significant event, contrasting with the proteasome-mediated degradation of β-catenin. GSN downregulates E-cadherin and β-catenin while upregulating N-cadherin. Tumor cells undergoing a cadherin switch, characterized by the loss of E-cadherin and the gain of N-cadherin expression, exhibit aggressive metastatic phenotypes^20^.

Understanding the significance of these genes in colorectal cancer provides valuable insights into the underlying molecular mechanisms that drive tumor progression, invasion, and metastasis. Targeting these genes or their associated signaling pathways holds potential as therapeutic strategies for colorectal cancer treatment, ultimately leading to improved patient outcomes. This study aimed to use bioinformatics approaches to detect cell adhesion prominent genes CDC42, TAGLN, and GSN in colonic-polyps and examine the clinical significance of the genes as a prognostic biomarker in order to assess colon cancer risk. The findings provide a comprehensive analysis of the expression patterns of cell adhesion genes in high and low-risk colon polyps compared to normal adjacent tissues. The correlation between gene expression and immunophenotype, as well as the role of mi-RNAs and lncRNAs in gene overexpression, were also evaluated. Gene ontology (GO) and KEGG analysis were performed to identify pathways related to adhesion molecule gene expression in various tissues. This study will deepen our understanding of the molecular mechanisms of colon cancer and inform the development of improved personalized diagnostic, prognostic, therapeutic biomarkers in COAD cancer.

## Materials and methods

### Tissue collecting samples

This study employed a descriptive-analytical design to investigate a cohort of 20 patients diagnosed with colon polyps, along with 20 paired normal tissue samples serving as a control group. Participants were recruited through the Colorectal Cancer Screening Program and were referred to the gastrointestinal clinic for colonoscopy due to suspected clinical symptoms. Two expert pathologists, blinded to the study objectives, confirmed the pathologic features of the samples at Taleghani Hospital. The study adhered to the ethical guidelines outlined in the Declaration of Helsinki, including obtaining written consent from all human research participants. All procedures were conducted under the oversight of the Ethics Committee of the Shahid Beheshti University of Medical Sciences in accordance with the university’s policies on medical and research ethics.

### Reverse transcriptase PCR (RT-PCR)

Total RNAs were extracted from obtained samples (Yekta Tajhiz Azma kit, Cat No. YT9065, Teheran, Iran). The RNAs concentration was measured by Nanodrop (NanoDrop Technologies, Wilmington, DE, USA) for acceptable quantification and qualification. RNAs were converted to cDNA by Retro transcriptase (RT) reaction (Yekta Tajhiz Azma kit, Cat No. YT9065, Teheran, Iran) after normalization. The following reagents were added into a sterile nuclease-free tube on ice by order as mentioned: 0.1 ng of justified total extracted RNA were picked up and mixed with 1.0 µl of Random hexamer primer, then DEPC-treated water was added up to 13.5 µl. The ads in mixed gently, and incubated at 70 °C for 5 min as manufacturer protocol. Denaturation was performed at 95 °C for next 5 min and the following cDNA Synthesis Mix according to protocol was mixed up. The ingredients were as follow: 4 µl of 5 × first-strand buffer, 1 µl of dNTPs (10 mM each), 0.5 µl of Rnasin (40U/μl), and 1 µl M-MLV en zyme. The mixture was added to the denatured RNA and incubated for 60 min at 37 °C. Finally, the reaction was terminated by heating at 70 °C for 5 min. The synthesized cDNAs were kept at − 20 °C until use. Primers for the loading control GAPDH generated a PCR product of 131 bp. Gene-specific primers for TAGLN, GSN and CDC42 were designed to produce PCR products of 166, 64 and 147 bp, respectively. Primer sequences were as follows (for TAGLN, GSN and CDC42 specificity conferred by the 3′ end of the primer) (Table 1).

**Table 1:** Sequences of the forward (F) and reverse (R) primers used for Real-time PCR.

Gene	Gene ID	Primer sequence (F forward, R reverse)		Product size (bp)
*TAGLN*	6876	F	5′- AATGGCGTGATTCTGAGCAAGC-3′	166 bp
		R	5′- GGAACATGTCAGTCTTGATGACC-3′	
*GSN*	2934	F	5′- TGGGAGAGCTTCAACAATGGC-3′	64 bp
		R	5′- ACCACTGGTGGATGTTGTTGC-3′	
*CDC42*	998	F	5′- AAAGAAAAGTGGGTGCCTGAG-3′	147 bp
		R	5′- AGCAGTCTCTGGAGTGATAGG-3′	
*GAPDH*	2597	F	5′- GTCTCCTCTGACTTCAACAGCG-3′	131 bp
		R	5′- ACCACCCTGTTGCTGTAGCCAA-3′	

### Real time-PCR

Following cDNA synthesis, *CDC42*, *TAGLN*, and *GSN* genes expression level was evaluated by Real-time PCR via SYBER Green qPCR method. The quantification was performed by use of MasterMix 2x (Yektatajhiz kit, Cat No YT2551, Tehran, Iran). Real-time PCR was carried out by designed primers as mentioned. The justification of the primers was estimated by Linreg method and the efficiency of each primer was calculated 95%. The Real time reactions were administered under the following condition: pre-denaturation at 95 °C for 20 s in one cycle, denaturation at 95 °C for 10 s, annealing on 60 °C for 10 s, extension on 72 °C for 20 s, 40 cycles repeat all three main levels. Amplification signals were normalized by *glyceraldehyde 3-phosphate dehydrogenase* (*GAPDH*) as house-keeping gene. The relative quantification and fold changes were evaluated by the 2^−ΔΔCt^ method^14^.

### Real time-PCR statistical analysis

All released RT-PCR data were analyzed by GraphPad Prism version 9 software (San Diego, CA, USA). The data were not-normally distributed and the non-parametric tests were applied. The Mann Whitney and between-groups comparisons were tested for statistical significance using the paired t-test and one-way analysis of variance (ANOVA), respectively. All *P*-values < 0.05 were considered statistically significant.

### Adhesion molecule genes correlation in healthy tissue

To investigate the co-expression of *CDC42*, *TAGLN*, and *GSN* genes at the transcript level in healthy colon tissue, the “correlation analysis” tool from the GEPIA2 database (http://gepia2.cancer-pku.cn) and the Pearson correlation coefficient method were utilized. The correlation of gene expression was evaluated one by one in healthy sigmoid and transverse colon samples^15^.

### Transcription factors regulating adhesion molecule genes

According to the significant difference in the expression of *CDC42*, *TAGLN*, and *GSN* genes in high-risk and low-risk polyps, the transcription factor regulating the target genes, as well as information on how these interactions are regulated, were extracted and analyzed from the TRUST database (https://www.grnpedia.org/trrust/)^16^.

### Adhesion molecules co-expressed genes and PPI network

GEPIA2 (http://gepia2.cancer-pku.cn) was employed to retrieve the 100 co-expression genes of each *CDC42*, *TAGLN*, and *GSN* gene in TCGA COAD tumor, TCGA adjacent normal tissues, and GTEx colon samples. Protein–protein interaction (PPI) network of CDC42, TAGLN, and GSN was displayed by the STRING database (http://string-db.org). The minimum required interaction score was set at 0.4. Then, nodes with edges < 1 and nodes not involved in the leading integrated network were excluded^15,17^.

### MicroRNAs regulating adhesion molecule genes

DIANA-microT database (http://diana.imis.athena-innovation.gr) was utilized to identify miRNAs regulating *CDC42*, *TAGLN*, and *GSN* genes. Common miRNAs identified by DIANA and validated by strong evidences from laboratory studies in the mirTarbase database and were considered potential gene regulatory miRNAs. In the Following, the Bioinformatics and Evolutionary Genomics web tool (https://bioinformatics.psb.ugent.be/webtools/Venn/) was employed to intersect the sets, including common miRNAs, common proteins in the PPI network, as well as their common co-expressed genes and draw the Venn diagram^18,19^.

### The relationship between miRNAs and lncRNAs in adhesion molecule genes

The miRNet database, a miRNA-centric network visual analytics platform (https://www.mirnet.ca/) was employed to identify lncRNAs related to adhesion molecule gene function. By use of experimentally validated miRNAs, we obtained a list of lncRNAs for each gene and analyzed their overlap using the Genomics web tool (https://bioinformatics.psb.ugent.be/webtools/Venn/)^19,20^.

### Adhesion molecule genes expression and survival analysis in COAD samples

To investigate the expression levels of adhesion molecule genes (*CDC42*, *TAGLN*, and *GSN*) in colon adenocarcinoma (COAD), we utilized the GEPIA2 web tool (http://gepia2.cancer-pku.cn). The expression analysis was performed by comparing retrieved COAD data from TCGA and healthy samples from GTEx. In addition, we used GEPIA2 to evaluate the relationship between adhesion molecule genes and survival in COAD patients using the clinical data and gene expression data from TCGA. The *P*-value cut-off for statistical significance was set at 0.05^15^.

### Adhesion molecule genes functional enrichment analysis

Further, using the Enrichr database (https://maayanlab.cloud/Enrichr/), Gene Ontology (GO) enrichment analysis and Kyoto Encyclopedia of Genes and Genomes enrichment analysis (KEGG) was performed using the aforementioned co-expressed genes for each of the three *CDC42*, *TAGLN*, and *GSN* genes in GTEx healthy colon, COAD adjacent normal, and COAD tissues. Then, records with *P*-value < 0.05 were selected and the top features-based *P*-value candidates were displayed by the “ggplot” package in the R programming language^21^.

### Investigating adhesion molecule genes expression in relation to immune subtypes of COAD cancer

The expression of adhesion molecule genes (*CDC42*, *TAGLN*, and *GSN*) was analyzed in relation to six immune subtypes of COAD cancer (C1-C6) using TISIDB, an integrated repository portal for tumor-immune system interactions (http://cis.hku.hk/TISIDB/index.php)^22^.

### Drug-adhesion molecule genes interaction

Drug-gene interactions of drugs that interact with DEGs were retrieved and analyzed using the Drug Gene Interaction Database (DGIdb) (https://www.dgidb.org/). The final list of drugs was prepared according to the drugs approved by the Food and Drug Administration (FDA) in the DrugBank database^23^.

### Ethics approval and consent to participate

This study was approved by the ethical committee (Ethics No. No.IR.IAU.PS.REC.1400.423) of the Islamic Azad University, Tehran, Iran. Informed consent was obtained from all participants included in the study.

## Results

### Clinicopathological characteristics of patients with pre-cancerous lesions

Clinicopathological features of the 20 included patients with colon polyps were extracted from their files and presented at the beginning of the survey. All resected samples were categorized into high-risk and low-risk polyps based on reported pathological feature of the samples. The mean age of the patients was (60.32 ± 10.17 years). The analyzed data was presented in Table 2. The 43-years-old woman was the youngest and the 84 years-old man was the oldest patient who entered the study. The high-risk polys were detected in more than 56.3% of over 50 years-old patients. Most of male patients (60%) were distinguished with high-risk polys in contrast with under 50% of females reported with invasive polyps. Although 75% of patients with high-risk polyps verified positive family history of colon diseases but no significant P-value was admitted to familial history. In present survey, polyps detected in proximal-right colon (including the cecum, ascending colon, transverse colon) were significantly (*P*-value < 0.022) classified in high-risk category. The mean of polyps’ size was (14.21 ± 12.73 mm). As we desired, more than 90% of large polyps (≥ 10 mm) were identified with high-risk pathology report (*P* value < 0.001).

**Table 2:** Descriptive analysis of clinicopathological characteristics based on the histology of polyps.

Characteristic		High-risk polyp	Low-risk polyp	*P*-value
Age (years, mean ± SD)				0.99
< 50	(Min: 43, Max:84) 60.32 ± 10.17	50%	50%	
> 50		56.3%	43.8%	
Sex				0.617
Male	25%	60%	40%	
Female	75%	40%	60%	
Family history of colorectal cancer				0.197
Yes	40%	75%	25%	
No	60%	41.7%	58.3%	
Location of adenomas				0.022*
Proximal	55%	81.8%	18.2%	
Distal	45%	22.2%	77.8%	
Size of adenoma (mm, mean ± SD)				0.001*
< 10 mm	(Min:4,Max:50) 14.21 ± 12.73	11.1%	88.9%	
$\ge$ 10 mm		90.9%	9.1%	
Dysplasia				0.000*
High-grade	40%	100%	0%	
Low-grade	60%	18.2%	81.8%	

*Significant P-value.

### Selected cell adhesion genes CDC42, TAGLN and GSN expression in pre-cancerous lesions

The mRNA expression of desired genes was measured by Real-time PCR technique and the relative quantification (RQ) of each gene was calculated via 2^−ΔΔCt^ method. The expression analyzed data were subdivided to over and under RQ = 1 to represent up and down regulation of genes. The confirmed significant clinicopathological parameters were evaluated with nominated genes expression analysis. It is inevitable that upregulated genes recognized with large polyp size (≥ 10 mm) but no significant *P*-value was observed in this survey. Down regulation of *CDC42* was observed in small polyps (< 10 mm). The up regulation of *CDC42* and *TAGLN* were significantly related with high-grade dysplasia. The polyps with high-grade dysplasia displayed the up regulation in *GSN* but the *P-*value was not significant. Down regulation of *CDC42* in distal colon with 66.7% of cases and up regulation in proximal with 75.7% was seen in SPSS analysis with *P-*value < 0.017. More than 72% of cases with upregulated *TAGLN* was seen in proximal colon and only 27% of polyps in proximal was down regulated in *TAGLN* (Table 3).

**Table 3:** Association of mRNA expression of target genes with clinicopathological features in polyps.

mRNA expression		Parameters								
		Size			Dysplasia			Location		
Genes	RQ^#^	< 10 mm	≥ 10 mm	*P-*value	High-grade	Low-grade	*P-* value	Proximal	Distal	*P-*value
*CDC42*	< 1	66.7%	27.3%	0.17	11.1%	60.7%	0.010*	24.3%	66.7%	0.017*
	> 1	33.3%	72.7%		88.9%	39.3%		75.7%	33.3%	
*TAGLN*	< 1	55.6%	18.2%	0.16	11.1%	54.5%	0.03*	27.3%	44.4%	0.64
	> 1	44.4%	81.8%		88.9%	45.5%		72.7%	55.6%	
*GSN*	< 1	44.4%	36.4%	0.99	33.3%	45.5%	0.67	36.4%	44.4%	1.00
	> 1	55.6%	63.6%		66.7%	54.5%		63.6%	55.6%	

^#^Relative quantification, *Significant P-value.

### Expression level of CDC42, TAGLN and GSN in high-risk and low-risk polyps

**Figure 1:**
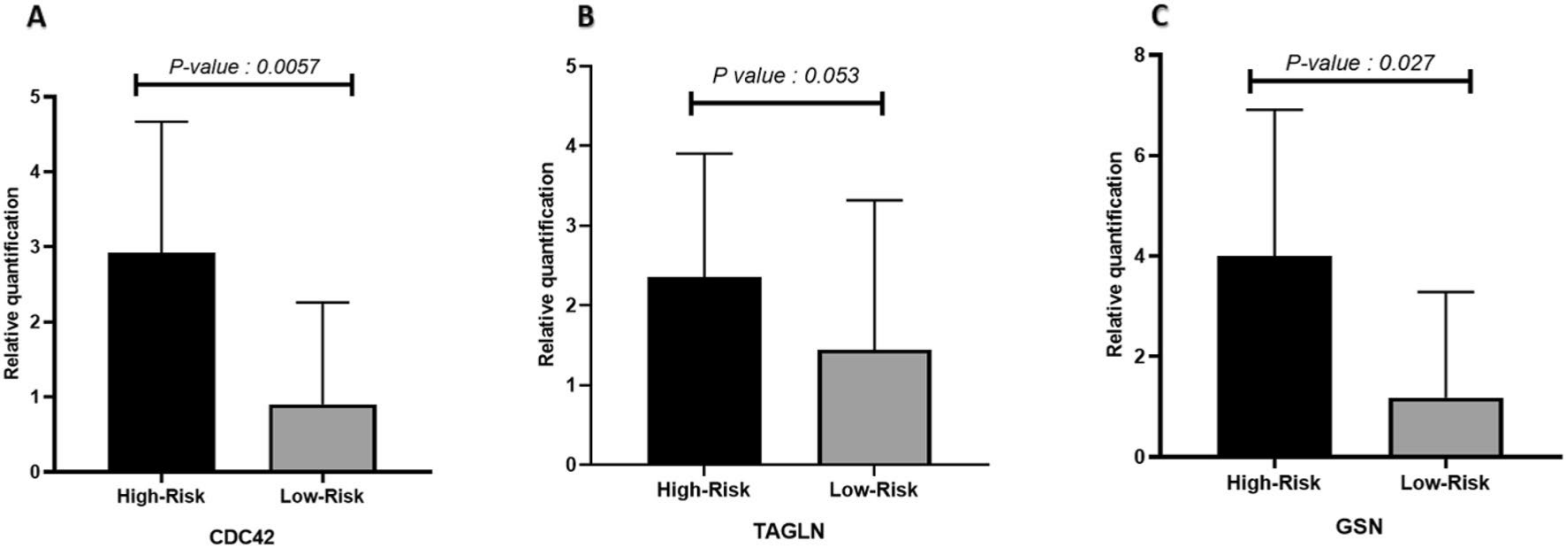
Expression levels of *CDC42, TAGLN,* and *GSN* in relation to the risk of malignancy in polyps.

Comparing the expression level of *CDC42, TAGLN,* and *GSN* based on the risk of malignancy in polyps showed a diminished expression level of *CDC42* in low-risk polyps in contrast with high-risk cases (*P-value* = 0.0057). The expression level difference between high-risk and low-risk categorized samples type in *TAGLN* was near significant with up regulation preference in high-risk lesions (*P-value* = 0.053). The third gene, *GSN*, admitted the significant up regulation of high-risk samples in contrast with low-risk expression levels showed substantial differences between polyp types (*P-value* = 0.027) (Fig. 1).

### Tests of between-subjects effects based on variable of histology

Between-subject effects based on variable of histology were tested using analysis of variance (ANOVA). Results showed significant effects in polyp size (P = 0.006), location (P = 0.006), and dysplasia (P = 0.000). Additionally, ANOVA analysis revealed significant expression levels of CDC42 and TAGLN in polyp histology (P = 0.006 and 0.005, respectively) (Table 4).

**Table 4:** ANOVA results for between-subjects effects based on variable of histology.

Descriptive		Mean ± STD	95% confidence interval for Mean		F	*P*-value
			Lower bound	Upper bound		
Sex	High risk	1.18 ± 0.40	0.91	1.45	0.56	0.46
	Low risk	1.33 ± 0.50	0.95	1.72		
Age	High risk	1.82 ± 0.40	0.12	1.55	0.04	0.83
	Low risk	1.78 ± 0.44	0.14	1.44		
FH	High risk	1.55 ± 0.52	1.19	1.90	2.17	0.15
	Low risk	1.22 ± 0.44	0.88	1.56		
Size	High risk	1.91 ± 0.30	1.71	2.11	31.55	0.000*
	Low risk	1.11 ± 0.33	0.85	1.37		
Location	High risk	1.18 ± 0.40	0.91	1.45	9.91	0.006*
	Low risk	1.78 ± 0.44	1.44	2.12		
Dysplasia	High risk	1.82 ± 0.40	1.55	2.09	36.45	0.000*
	Low risk	1.00 ± 0.00	1.00	1.00		
*CDC42*	High risk	1.82 ± 0.40	1.55	2.09	9.91	0.006*
	Low risk	1.22 ± 0.44	0.88	1.56		
*TAGLN*	High risk	1.91 ± 0.30	1.71	2.11	10.15	0.005*
	Low risk	1.33 ± 0.50	0.95	1.72		
*GSN*	High risk	1.73 ± 0.46	1.41	2.04	1.61	0.22
	Low risk	1.44 ± 0.52	1.04	1.85		

*Significant P-value. *STD* standard deviation.

### Prognostic biomarker determination in high-risk and low-risk pre-cancerous lesions

**Figure 2:**
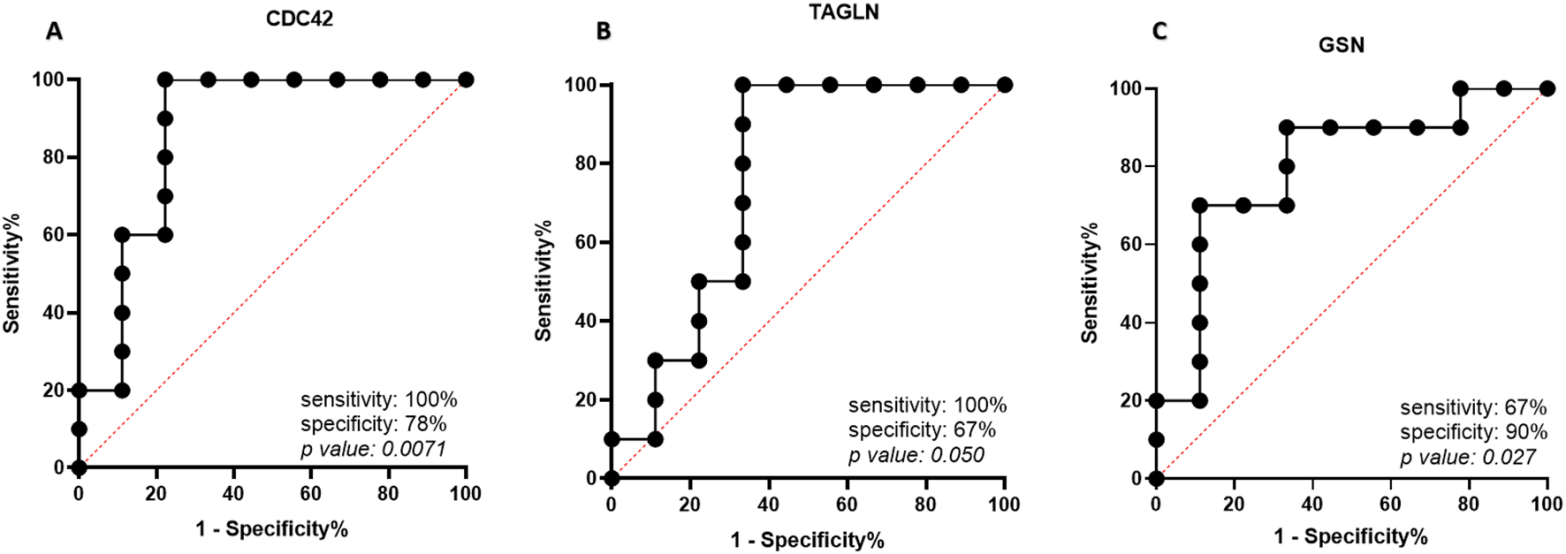
ROC curves showing AUC, sensitivity, and specificity of selected genes as biological biomarkers.

We evaluated the biomarker performance of the selected genes based on their sensitivity, specificity and the area under the curve (AUC). The receiver operating curve (ROC) was plotted for selected cell adhesion genes, their corresponding optimal cut-offs and AUC values were calculated. The predictive value of *CDC42, TAGLN,* and *GSN* expression in patients with high-risk and low-risk polyps were determined by ROC curves analysis. The AUC for *CDC42* was evaluated 0.87 by sensitivity of 100% and specificity of 78% (*P-value* = 0.0071). The *TAGLN* expression with 77% area under the curve demonstrates sensitivity of 100% and specificity of 67% with significant *P-*value to detect between high-risk and low-risk polyps. (*P-value* = 0.050) The estimated area for *GSN* ROC was 0.80 with sensitivity of 67% and specificity of 90% (*P-value* = 0.027) (Fig. 2).

### Correlation of CDC42, TAGLN, and GSN genes expression in healthy colon

**Figure 3:**
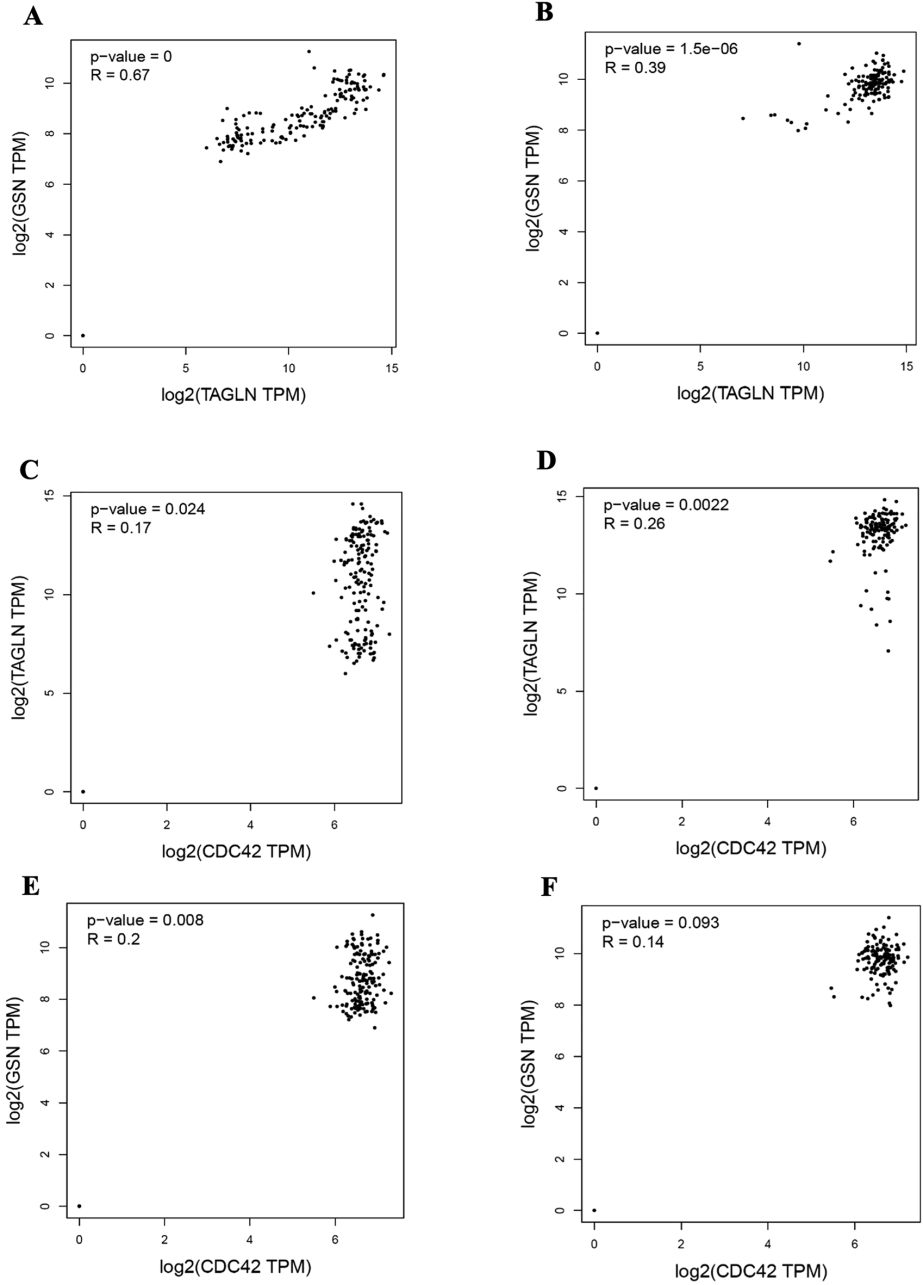
Correlation of *CDC42, TAGLN,* and *GSN* genes expression. (**A**) The correlation of TAGLN and GSN at transcriptome level in GTEx transverse colon. (**B**) The correlation of TAGLN and GSN at transcriptome level in GTEx sigmoid colon. (**C**) The correlation of CDC42 and TAGLN at transcriptome level in GTEx transverse colon. (**D**) The correlation of CDC42 and TAGLN at transcriptome level in GTEx sigmoid colon. (**E**) The correlation of CDC42 and GSN at transcriptome level in GTEx transverse colon. (**F**) The correlation of CDC42 and GSN at transcriptome level in GTEx sigmoid colon.

To validate the co-expression of *CDC 42, TAGLN,* and *GSN* at the transcript level in healthy sigmoid and transverse colon data from GTEx, the “correlation analysis” tool based on RNA-seq data and Pearson’s correlation coefficient from the GEPIA2 database was executed. According to the obtained results, the highest correlation was seen between *TAGLN* and *GSN* genes. So that their expression in the transverse colon and sigmoid colon samples showed a strong significant positive correlation in transverse colon (*P-value* = 0.00 and R = 0.67) and moderate significant positive correlation (*P*-value = 1. 5*10^-6 and R = 0.39) at the transcript level, respectively in sigmoid. *CDC42* gene had a weaker expression correlation with *TAGLN* and *GSN* genes. So that *CDC 42* and *TAGLN* had a negligible correlation in the transverse colon (*P*-value = 0 and R = 0.17), and in the sigmoid colon tissue, a weak positive expression correlation (*P*-value = 0.024 and R = 0.26) was recorded. On the other hand, the correlation between the expression of *CDC42* and *GSN* genes in the sigmoid colon was not significant (*P-value* = 0.09 and R = 0.14), but there was a weak positive correlation in the transverse colon (*P*-value = 0.008 and R = 0.2) (Fig. 3).

### Transcription factors targeting CDC42, TAGLN, and GSN Genes

Considering the significant difference in the expression of *CDC 42*, *TAGLN*, and *GSN* genes in high-risk and low-risk polyps, we investigated these genes’ possible transcription factors using the TRRUST database. We found that PTTG1 (PTTG1 Regulator of Sister Chromatid Separation, Securin) as a critical transcription factor is associated with the regulation of *CDC42* expression. It was also shown that three transcription factors, SRF (Serum Response Factor), MKL1, and KLF4 (KLF Transcription Factor 4), target the *TAGLN* gene and are effective in regulating the expression of this gene. Despite these two mentioned genes, the *GSN* gene was not found in the TRRUST database; therefore, there is no information about the transcription factors influential in the expression of this gene.

### Co-expressed genes of CDC42, TAGLN, and GSN in COAD and healthy samples

In order to investigate and compare the co-expression of adhesion molecules (*CDC42*, *TAGLN*, and *GSN*) in cancerous and healthy samples, 100 co-expressed genes were retrieved from GEPIA2 using data from TCGA COAD tumor samples, TCGA adjacent normal tissue samples, and GTEx healthy colon samples. The analysis of these genes revealed several notable findings.

**Figure 4:**
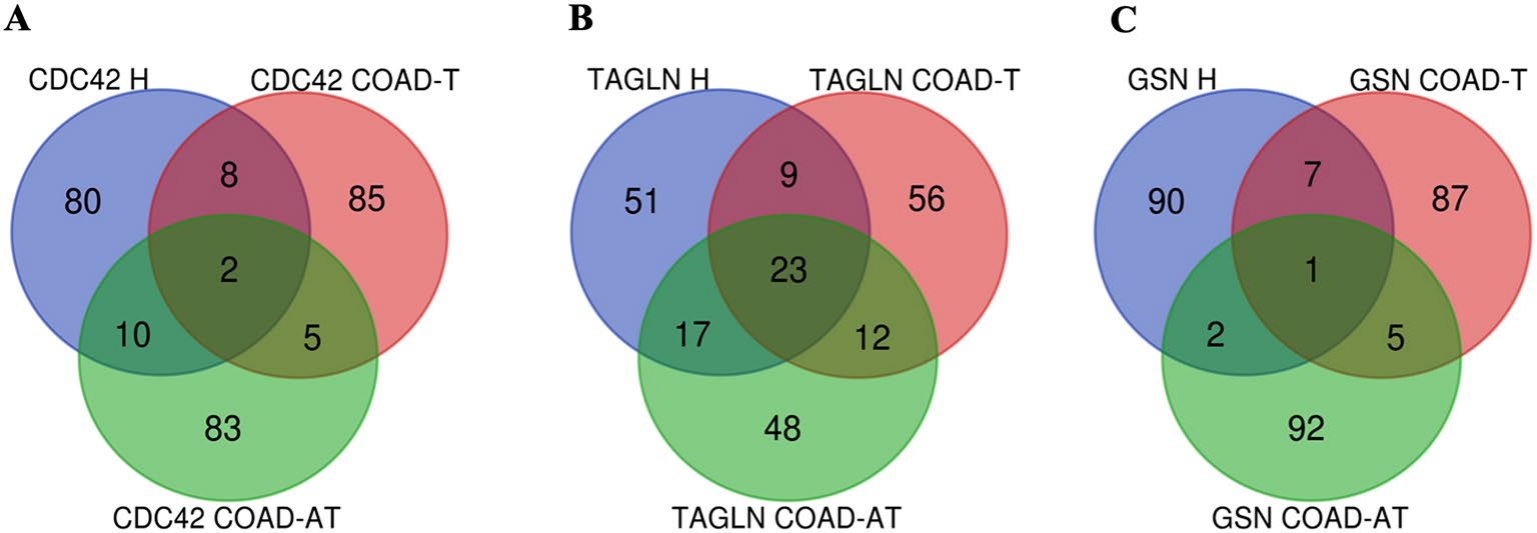
Intersection of adhesion molecules (*CDC42*, *TAGLN*, and *GSN*) co-expressed genes in healthy colon tissue (blue), adjacent normal tissue (green), and COAD cancer tissue (red). (**A**) intersection of *CDC42* co-expressed genes. (**B**) intersection of TAGLN co-expressed genes. (**C**) intersection of *GSN* co-expressed genes. *CDC42* H: CDC42 co-expressed genes in healthy colon, *CDC42* COAD-AT: *CDC42* co-expressed genes in adjacent normal tissue, *CDC42 COAD-T*: *CDC42* co-expressed genes in COAD tissue, *TAGLN H*: *TAGLN* co-expressed genes in healthy colon, *TAGLN* COAD-AT: *TAGLN* co-expressed genes in adjacent normal tissue, *TAGLN* COAD-T: *TAGLN* co-expressed genes in COAD tissue, *GSN* H: *GSN* co-expressed genes in healthy colon, *GSN* COAD-AT: *GSN* co-expressed genes in adjacent normal tissue, *GSN* COAD-T: *GSN* co-expressed genes in COAD tissue.

In the case of *CDC42*, the *TWF1* and *SELT* genes were identified as co-expressed in all three groups: healthy tissue, adjacent healthy tissue, and tumor tissue. Additionally, ten genes, including *CYB5R4, ATG5, SLC25A46, CNIH1, PSMA6, SNX6, TXNDC9, ARPC4, PDCD10,* and *UBE2N*, were found to be co-expressed in both healthy tissue and adjacent normal tissue. In comparison, eight genes, including *GNAI3, WSB2, BZW1, LUZP1, CAP1, SH3GLB1, CHMP2B,* and *VAPA*, were co-expressed in both healthy tissue and tumor tissue. Lastly, five genes including *GSKIP, SELENOF, RAP1B, UBE2D3,* and *GTF2B*, were found to be co-expressed in both adjacent healthy tissue and tumor tissue (Fig. 4A).

In the case of *TAGLN*, an analysis of the obtained genes revealed that 23 genes, including *FERMT2, CFL2, ACTA2, HSPB6, AOC3, LDB3, AP000892.6, CASQ2, HSPB8, ACTG2, DNAJB5, CSRP1, JPH2, MYL9, KCNMB1, DES, TPM2, FLNC, FLNA, CNN1, PDLIM3, DACT3,* and *TGFB1I1* were commonly present in all three groups: healthy tissue, adjacent healthy tissue, and tumor tissue. Additionally, 17 genes, including *SPEG, VCL, ILK, ACTN1, CPXM2, LOXL4, PTRF, TUBA1A, JAM3, NCS1, TSPAN2, PDLIM7, PTGES3L, CTXN1, TPM1, LGALS1,* and *SMTN* were found to be co-expressed in healthy and adjacent healthy tissue groups. Furthermore, nine genes, including *TNS1, PPP1R14A, MSRB3, SYNPO2, MRGPRF, SYNM, MYH11, ATP2B4,* and *MRVI1*, were co-expressed in healthy and tumor tissue groups, and 12 genes, including *PYGM, FBXL22, C8orf88, BVES, LMOD1, CPEB1, ATP1A2, PDZRN4, FILIP1, ANGPTL1, MYLK*, and *ARHGEF25* were found to be co-expressed in the group of adjacent healthy tissue and tumor tissue (Fig. 4B).

Lastly, the *GSN* gene was also analyzed by comparing and identifying common co-expressed genes among healthy tissue, adjacent healthy tissue, and tumor tissue groups. The *TIMP2* gene was found to be present in all three groups. The *TCF7L1* and *PTPRM* genes were also identified as co-expressed in the healthy and adjacent normal tissue groups. Moreover, five genes, including the *SLC12A4, SLIT3, GYPC, TSPAN4, CYYBRD1, CYB5R3*, and *FBLN5* were identified as co-expressed in the healthy and tumor tissue groups. Lastly, the *EHD2, CTIF, FBXO32, PIP5K1C,* and *AHNAK* genes were found to be co-expressed in the adjacent healthy tissue and tumor tissue groups (Fig. 4C).

### PPI network of adhesion molecule genes

**Figure 5:**
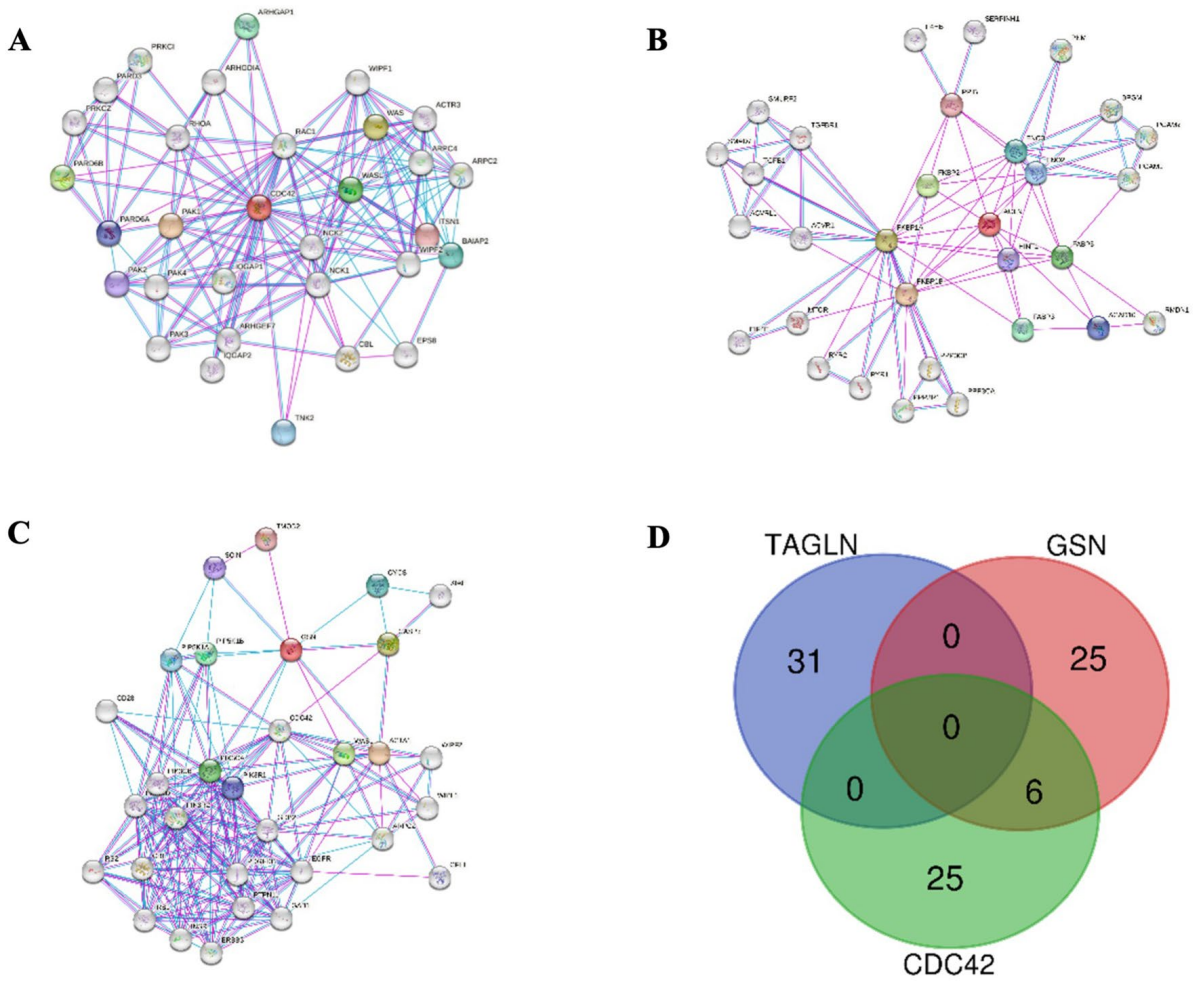
PPI network of *CDC42*, *TAGLN*, and *GSN* genes. (**A**) PPI network of 30 CDC42-binding proteins. (**B**) PPI network of 30 TAGLN-binding proteins. (**C**) PPI network of 30 *GSN*-binding proteins. (**D**) Intersection analysis of proteins present in *CDC42*, *TAGLN*, and *GSN* genes PPI networks.

We also retrieved the PPI network, including the top 30 proteins associated with *CDC42*, *TAGLN*, and *GSN* genes from STRING, which were supported by experimental data. Figure 5 shows the interaction network of the mentioned genes and proteins. Furthermore, in order to identify common proteins among these adhesion prominent genes and to find out the expected functional mechanism of selected genes, the proteins present in the PPI network of genes were intersected, which is displayed in Fig. 5D. Intersection analysis of PPI networks illustrated those six proteins, including ARPC2, WASL, WIPF2, CBL, CDC42, and WIPF1, which are shared among the PPI network of *CDC42* and *GSN* genes. This is while no common proteins were found among the PPI network of the *TAGLN* gene and the two other explored genes.

### miRNAs contributed to adhesion molecule genes expression

We attempted to identify miRNAs regulating prominent cell adhesion genes *CDC42*, *TAGLN* and *GSN*. DIANA and mirTarbase databases were used to identify miRNAs regulating these genes. The data extracted from DIANA, considering the miTG score > 0.7, indicates the presence of 141 potential miRNAs regulating *CDC42*. In comparison, the mirTarbase database reports the presence of 24 potential miRNAs regulating *CDC42*. By intersecting the data of the two databases, seven common miRNAs were obtained; among these miRNAs, hsa-miR-29a-3p, hsa-miR-29b-3p, hsa-miR-137, hsa-miR-185-5p, hsa-miR-29c-3p have been validated by strong laboratory evidence. In addition, although miRNAs such as hsa-miR-204-5p, hsa-miR-133b, hsa-miR-216a-5p, hsa-miR-330-3p, hsa-miR-608, hsa, miR-224-5p, hsa-miR -195-5p, hsa-miR-133a-3p, hsa-miR-375 were not present in the miRNAs obtained from DIANA, but they have been validated by strong laboratory evidence and are among the known miRNAs regulating *CDC42* expression.

Regarding the *TAGLN* gene, 13 and 29 miRNAs regulating gene expression were obtained from DIANA and mirTarbase databases, respectively. Through the investigation, it was found that there was no common miRNA in the data of the two databases, and no miRNA was validated by strong laboratory evidences. Similar results were obtained in the investigation of miRNAs regulating the *GSN* gene. So that 22 and 32 potential regulatory miRNAs were acquired from DIANA and mirTarbase databases, respectively, and there was no commonality between them. However, in the outcomes obtained from mirTarbase data, it was encountered that strong laboratory evidence supports the regulatory role of hsa-miR-141-3p in *GSN* gene expression.

In order to identify common co-expression mechanisms in increasing the expression of prominent cell adhesion genes, miRNAs obtained from different databases were intersected. The results obtained from intersecting DIANA data demonstrated that hsa-miR-423-5p is common between *GSN* and *CDC42* genes, and hsa-miR-4498 is shared between *GSN* and *TAGLN* genes. This is even though no commonality among the presented miRNAs of *CDC 42* and *TAGLN* genes. Meanwhile, intersecting the results of mirTarbase reveals the common presence of hsa-miR-1-3p among *CDC42* and *TAGLN* genes. In addition, more common miRNAs, including hsa-miR-6127, hsa-miR-6129, hsa-miR-4419-a, hsa-miR-4510, hsa-miR-6130, hsa-miR-6133 were illustrated among genes *GSN* and *TAGLN* genes. In mirTarbase data, there was no commonality between *GSN* and *CDC42* genes. Therefore, it can be concluded that *GSN* and *TAGLN* genes have stronger expression correlations among the three discussed prominent cell adhesion genes.

### lncRNAs targeted by adhesion molecule genes regulating miRNAs

To comprehend the functional genetic mechanisms involved in developing high-risk adenoma polyps and the role of adhesion molecule genes, we examined target lncRNAs and their corresponding miRNAs. Given a large number of miRNAs, we focused on those validated through experimental tests previously. Using the miRNet database and 11 miRNAs reported for the *CDC42* gene, we identified 447 lncRNA targets. After reviewing and sharing the lncRNA data, we found that 217 targets were unique. The most common were KCNQ1OT1, NEAT1, XIST, HCG18, and OIP5-AS1, shared among 10, 9, 8, 7, and 6 *CDC42* miRNA targets, respectively.

Additionally, to identify the target lncRNAs of the *GSN* gene miRNAs, we evaluated hsa-mir-141-3p and obtained 46 lncRNAs. Twenty-five of these lncRNAs overlapped with the *CDC42* gene miRNA target lncRNAs, including PSMD6-AS2, MIR29B2CHG, SNHG16, KCNQ1OT1, XIST, PDCD4-AS1, TTN-AS1, EBLN3P, TUG1, MYLK-AS1, ZNF674-AS1, ZEB1-AS1, DSCAM-AS1, PRKCZ-AS1, SP2-, AS1, DNAAF4-CCPG1, NEAT1, MZF1-AS1, RPARP-AS1, MALAT1, SNHG15, OIP5-AS1, PTOV1-AS1, CCDC18-AS1, TMEM147-AS1. Further, due to the lack of validated miRNA reports for the *TAGLN* gene, subsequent lncRNA analysis was not conducted.

### Gene expression and survival analysis in COAD samples

**Figure 6:**
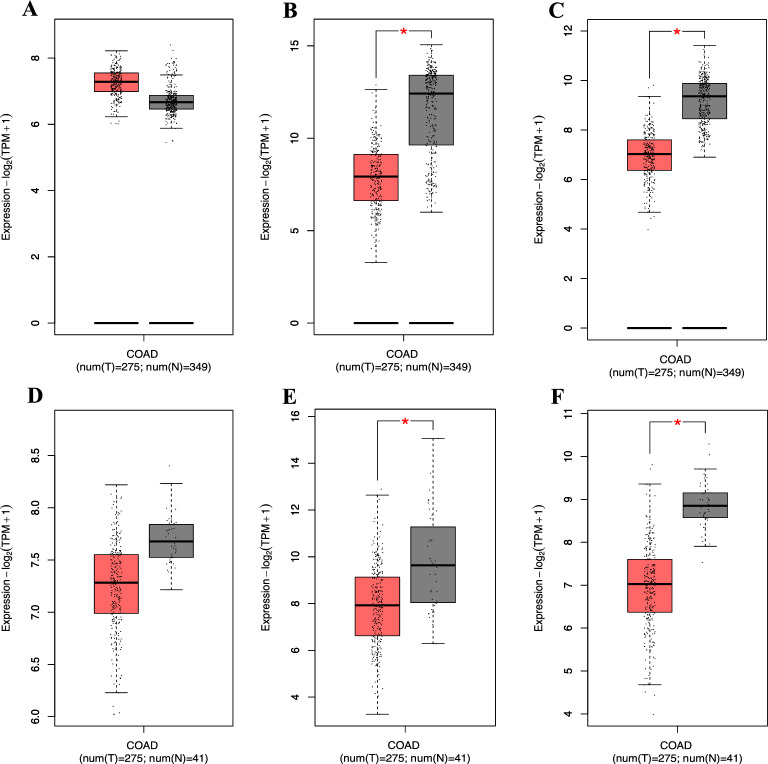
Gene expression of adhesion molecule genes in COAD patients compared to healthy samples and adjacent normal tissues. (**A**) Gene expression of *CDC42* gene in COAD patients compared to healthy samples. (**B**) Gene expression of *TAGLN* gene in COAD patients compared to healthy samples. (**C**) Gene expression of *GSN* gene in COAD patients compared to healthy samples. (**D**) Gene expression of *CDC42* gene in COAD patients compared to adjacent normal tissues. (**E**) Gene expression of *TAGLN* gene in COAD patients compared to adjacent normal tissues. (**F**) Gene expression of *GSN* gene in COAD patients compared to adjacent normal tissues. (*P-value < 0.05, **P-value < 0.01, ***P-value < 0.001).

The expression levels of adhesion molecule genes in colorectal adenocarcinoma (COAD) samples were analyzed using GEPIA2. The dataset comprised gene expression data from 275 COAD patients, 41 adjacent normal tissue samples, and 349 healthy tissue samples. The results showed a decrease in the expression of all three genes in cancer samples compared to high-risk colorectal adenoma. Figure 6B,C,E and F indicate a significant reduction in *TAGLN* and *GSN* genes expression in cancer samples compared to the healthy colon and adjacent normal tissues. However, there was no significant difference in the expression of the *CDC42* gene between cancer samples and healthy colon and adjacent normal tissue (Fig. 6A and D).

**Figure 7:**
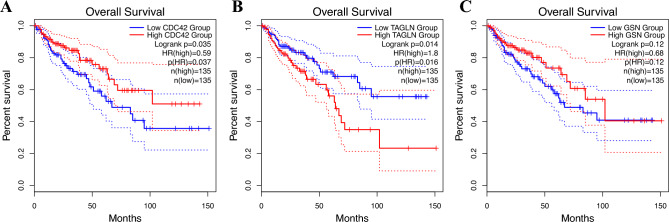
Kaplan–Meier survival analysis of adhesion molecule genes in COAD patients. The X-axis represents time in months, and the Y-axis represents the probability of survival. (**A**) Kaplan–Meier survival analysis of *CDC42* gene in COAD patients. (**B**) Kaplan–Meier survival analysis of *TAGLN* gene in COAD patients. (**C**) Kaplan–Meier survival analysis of *GSN* gene in COAD patients.

Survival analysis was conducted to investigate the relationship between the expression of adhesion molecule genes and survival rates in COAD patients. The results showed that while the expression level of the *GSN* gene had no significant relationship with survival rate (Fig. 7C), the expression of *CDC42* and *TAGLN* genes did have a meaningful relationship. However, their relationship with survival rate was opposite. Specifically, decreased expression of the *TAGLN* gene was associated with survival extension (*P-value* = 0.014) (Fig. 7B), while increased expression of the *CDC42* gene was associated with improved survival in COAD patients (*P-value* = 0.035) (Fig. 7A).

### Functional enrichment analysis of adhesion molecule genes in COAD and healthy tissues

Gene ontology (GO) and the KEGG pathway are widely used tools for analyzing gene function and biological pathways. They provide insights into the biological processes, molecular functions, and cellular components associated with specific genes. We analyzed the gene ontology and KEGG pathway of the adhesion molecule genes and their co-expressed genes in the healthy colon, adjacent normal tissue, and COAD cancerous tissues.

In this regard, studies have shown that in healthy colon tissue, *CDC42* and its co-expressed genes are related to various KEGG pathways such as 'endocytosis', 'Salmonella infection', 'adherens junction', and others. In terms of GO biological process, they are involved in 'protein transport', 'Golgi organization', and 'intracellular protein transport'. Their molecular functions include 'cadherin binding', 'guanyl ribonucleotide binding', and 'GTPase activity', while cellular components include 'organelle bounding membranes', 'focal adhesion', and 'secretory granule membrane'. In adjacent normal tissue, *CDC42* and co-expressed genes are linked to KEGG pathways such as 'oxidative phosphorylation', 'non-alcoholic fatty liver disease', 'Huntington's disease', and others. In terms of GO biological process, they play roles in 'aerobic electron transport chain', 'mitochondrial ATP synthesis', and 'NADPH dehydrogenase complex assembly'. Their molecular functions include 'oxidoreduction-driven active transmembrane transporter activity', 'NADH dehydrogenase ubiquinone activity', and others. Cellular components include 'mitochondrial inner membrane', 'organelle inner membrane', and 'mitochondrial respiratory chain complex I'. In COAD cancerous tissue, *CDC42* and co-expressed genes are linked to KEGG pathways such as 'endocytosis', 'chemokine signaling pathway', and 'leukocyte transendothelial migration'. In the GO biological process, they are involved in the 'regulation of lamellipodium assembly', 'regulation of cytokines', and 'actin-filament capping'. Their molecular functions include 'GTPase activity', 'nucleoside triphosphate activity', and 'cadherin binding', while cellular components include 'vesicles', 'endoplasmic reticulum membrane', and 'heterotrimeric G-protein complex'.

*TAGLN* and its co-expressed genes play roles in various biological processes, molecular functions, and cellular components in different tissue samples. In healthy colon tissue, they are linked to KEGG pathways such as 'focal adhesion', 'vascular smooth muscle contraction', 'tight junction', and others. In terms of GO biological process, they are involved in 'muscle contraction', 'platelet aggregation', and 'cell–matrix adhesion'. Their molecular functions include 'actin binding', 'alpha-actinin binding', and 'vinculin binding', while cellular components include 'focal adhesion', 'cytoskeleton', and 'collagen containing ECM'. In adjacent normal tissues, *TAGLN* and co-expressed genes are involved in KEGG pathways such as 'focal adhesion', 'hypertrophic cardiomyopathy', and 'tight junction'. In the GO biological process, they play roles in 'muscle contraction', 'platelet aggregation', and 'homotypic cell–cell adhesion'. Their molecular functions include 'actin binding', 'alpha-actinin binding', and 'collagen binding', and cellular components include 'focal adhesion', 'cell-substrate junction', 'cytoskeleton', and 'adherens junction'. In COAD cancerous tissues, *TAGLN* and co-expressed genes are associated with KEGG pathways such as 'vascular smooth muscle contraction', 'regulation of actin cytoskeleton', and 'cGMP-PKG signaling pathway'. In terms of GO biological process, they are involved in 'muscle contraction', 'regulation of muscle contraction', and 'smooth muscle contraction'. Their molecular functions include 'actin binding', 'alpha-actinin binding', and 'cAMP binding', and cellular components include 'sarcolemma', 'cytoskeleton', and 'focal adhesion'.

Moreover, regarding the *GSN* gene, it has been revealed that in healthy colon tissue, the gene *GSN* and its co-expressed genes participate in KEGG pathways such as 'adherens junction', 'proteoglycans in cancer', 'tight junction', and 'focal adhesion'. Their GO biological processes involve 'regulation of metallopeptidase activity', 'elastic fiber assembly', and 'cell–cell adhesion via the plasma membrane'. The molecular function of *GSN* and co-expressed genes includes 'metallopeptidase inhibitor activity', 'kinase binding', and 'protein tyrosine phosphatase activity'. The cellular component involves 'focal adhesion', 'cell-substrate junction', and 'integral component of plasma membrane'.

In adjacent normal tissue, *GSN* and its co-expressed genes participate in 'focal adhesion' and 'regulation of actin cytoskeleton' KEGG pathways. The biological processes they are involved in are 'cell junction assembly' and 'actin filament capping'. The molecular function involves 'nitric-oxide synthase binding', 'vinculin binding', and 'actin binding'. The cellular component involves 'focal adhesion', 'cell-substrate junction', and 'adherens junction'.

In COAD cancerous tissue, *GSN* and its co-expressed genes participate in the 'FOXO signaling pathway' and 'endocytosis' KEGG pathways. The biological processes they are involved in are 'peptidyl serine autophosphorylation' and 'regulation of cellular response to oxidative stress'. The molecular function includes 'cytoskeleton-nuclear membrane anchor activity', 'GTPase activator activity', and 'cytokine receptor activity'. The cellular component involves the 'cytoskeleton', 'cell-substrate junction', 'focal adhesion', 'collagen-containing ECM', and 'actin cytoskeleton'.

### Associations between adhesion molecule genes expression and immune subtypes in COAD samples

**Figure 8:**
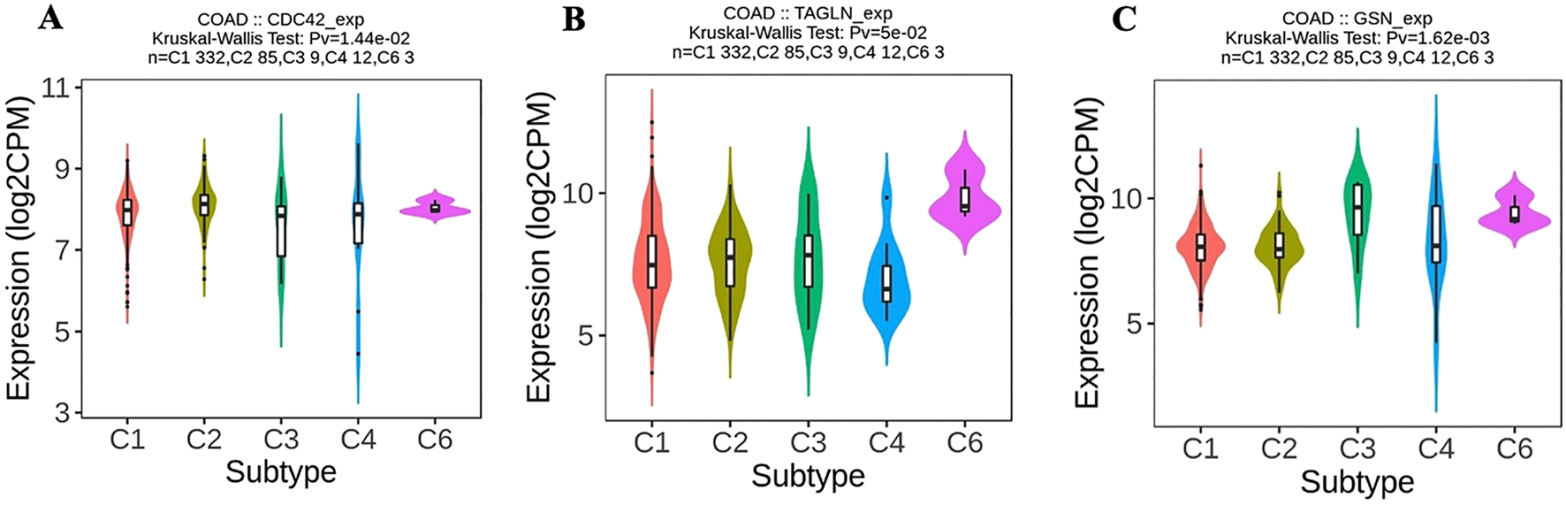
Associations between adhesion molecule genes expression and immune subtypes in COAD samples. (**A**) Associations between *CDC42* gene expression and immune subtypes in COAD samples. (**B**) Associations between *TAGLN* gene expression and immune subtypes in COAD samples. (**C**) Associations between *GSN* gene expression and immune subtypes in COAD samples.

Six subtypes of cancer have been identified based on the characteristics of the tumor microenvironment (TME) in various types of cancer^24^. These subtypes include C1 (wound healing), which has high levels of angiogenic genes, a high proliferation rate, and a Th2 cell bias in the adaptive immune infiltrate. C2 (IFN-g dominant) has the highest M1/M2 macrophage polarization, a strong CD8 signal, and high TCR diversity. C3 (inflammatory) is defined by elevated Th17 and Th1 genes, low to moderate tumor cell proliferation, and lower levels of aneuploidy. C4 (lymphocyte depleted) is found in specific subtypes of adrenocortical carcinoma, pheochromocytoma, paraganglioma, hepatocellular carcinoma, and gliomas. C5 (immunologically quiet) is mainly made up of brain lower-grade gliomas and exhibits the lowest lymphocyte and highest macrophage responses. C6 (TGF-b dominant) is a small group of mixed tumors with the highest TGF-b signature and a high lymphocytic infiltrate^24^. These subtypes suggest specific treatment approaches may be independent of histologic type. Most COAD patients belong to the C1 subtype^24^. To evaluate the correlation between adhesion molecule genes expression (*CDC42*, *TAGLN*, and *GSN*) and the immune subtypes of COAD samples, TISIDB has been utilized. The results indicate that *CDC42* gene expression is most strongly associated with the C2 subtype, followed by C1 (Fig. 8A). Studies also revealed that *TAGLN* has the highest expression correlation with the C6 subtype, followed by C2 and C3, while *GSN*'s highest expression correlation is with the C3 subtype, then C6 (Fig. 8B and C).

### Drug-gene interaction result

Examining the drug-gene interaction according to the results obtained from DGIdb, a total of 7 drugs were predicted that could potentially have a therapeutic effect on patients. Such drugs include SECRAMINE B, SECRAMINE A, CHEMBL563777, CHEMBL554475, CHEMBL562095, and GONADORELIN ACETATE, are effective on *CDC42* gene, and AZACITIDINE drug is effective on *TAGLN* gene. Among these drugs, only GONADORELIN ACETATE and AZACITIDINE have been approved. No drug-gene interaction was found for the *GSN* gene in the investigations.

## Discussion

Colorectal cancer (CRC) is a major public health concern, as it often lacks early-stage symptoms and contributes to a high mortality rate. In developing countries, effective strategies for cancer prevention have not been widely implemented^25^. Although surgical removal of tumors and chemotherapy are effective treatments, late diagnosis often hampers their impact on patient survival. Various risk factors for colon adenocarcinoma (COAD), including gene mutations, epigenetic changes, immune dysfunction, metabolic disorders, hormonal disorders, inflammation, H. pylori infection, and intestinal microbiota dynamics, have been identified^26^. Most colon cancers arise from polyps, some of which can become cancerous over time. Precision medicine may enhance the accuracy of COAD diagnosis, prognosis, and treatment response by analyzing gene expression changes. Consequently, researchers are examining gene expression alterations in CRC, including cell adhesion genes in COAD, to identify susceptibility biomarkers. However, studies investigating gene expression in polyps are limited. This study aimed to classify polyps as high-risk and low-risk to find a correlation between the overexpression of specific genes and dysplasia in high-risk polyps.

Our findings revealed that the increased expression of CDC42, TAGLN, and GSN in high-risk polyps may indicate a heightened risk of malignancy. We suggest that CDC42 and TAGLN could serve as potential biomarkers for polyp malignancy. TAGLN expression was lower in cancer samples compared to normal tissues, and CDC42 expression was negatively correlated with the prognosis of COAD patients. Decreased TAGLN expression was associated with survival extension.

CDC42 is known to play a crucial role in malignancies due to its involvement in various physiological functions, such as cytoskeletal and microtubule dynamics, transcription, cell cycle progression, cell polarity, apoptosis, phagocytosis, vesicle trafficking, and cell adhesion^27,28^. Our study found that CDC42 expression was significantly increased in high-risk polyp tissues, whereas its expression in COAD tissues was reduced compared to high-risk polyps. Moreover, higher CDC42 levels were correlated with improved survival outcomes for COAD patients. This discrepancy may be due to the different roles of CDC42 in polyp development and cancer progression. In high-risk polyps, the overexpression of CDC42 could contribute to dysplasia and neoplastic transformation. However, once the cancer has developed, the downregulation of CDC42 might be associated with other factors, such as the loss of cell polarity and the acquisition of invasive properties, leading to a worse prognosis. Further investigation is required to elucidate the precise role of CDC42 in these different stages of CRC development.

TAGLN, on the other hand, appeared to have a more complex relationship with COAD progression. Elevated TAGLN expression was associated with advanced COAD pathological stage and worse clinical outcomes, suggesting a driving role in COAD disease progression. We found that TAGLN expression was significantly increased in high-risk polyp tissues, yet a significant decrease in expression was observed in cancerous tissue compared to healthy and adjacent healthy tissue. This implies that higher TAGLN expression may contribute to polyp malignancy, but its role in cancer progression is not straightforward. It is possible that TAGLN overexpression in high-risk polyps promotes cell adhesion and the formation of precancerous tissue colonies, whereas its reduced expression in cancerous tissues may be related to alterations in its function, allowing cancer cells to acquire migratory and invasive properties.

The functional mechanisms of TAGLN and its co-expressed genes, as well as the immunophenotypes they contribute to, could explain the observed discrepancies. COAD patients often belong to the C1 immunosubtype (wound healing), which has a better prognosis. However, TAGLN expression is more closely associated with the less favorable C6 immunosubtype (TGF-b dominant). In addition, the functional enrichment analysis of co-expressed genes suggests that TAGLN's role may change from facilitating adhesion and preventing cell movement in healthy tissue to promoting cell migration and potential metastasis in cancerous tissue.

Previous studies have shown that TAGLN expression is regulated by TGFβ signaling and is activated in the late stages of COAD to induce a more aggressive phenotype^29,30^. In the early stages of tumor progression, TGFβ acts as an inhibitor of proliferation and migration; however, during advanced COAD and metastasis, it promotes tumor growth and invasion^31^. This dual role of TGFβ in COAD development and progression may explain the paradoxical observations regarding TAGLN expression.

Moreover, GSN, crucial actin-binding protein, is involved in various cellular functions, such as motility, shape, apoptosis, and phagocytosis. Although previous studies have reported changes in GSN expression in tumor cells compared to healthy cells, our study found no significant difference in GSN expression between colon polyps and normal tissues^32–37^. However, we did observe an increase in GSN expression in high-risk polyps compared to low-risk polyps. This suggests that GSN may play a role in polyp malignancy, but its impact on COAD progression remains unclear. Further studies are necessary to explore the involvement of GSN in the early stages of CRC development.

While our study's findings suggest the potential prognostic value of the CDC42, TAGLN, and GSN genes in colonic polyp lesions and colorectal cancer, it is essential to acknowledge certain limitations. Our study was based on a relatively small sample size, potentially affecting the representativeness of the heterogeneity of colonic polyp lesions and the generalizability of the results. Further validation studies involving larger cohorts are necessary to affirm the prognostic value of these genes. Another limitation arises from the reliance on correlation analysis between adhesion molecule gene expression and immunophenotype, utilizing TCGA data. While TCGA data is a valuable resource, it originates from different sources and platforms, introducing potential biases and variations. Caution must be exercised when interpreting these correlations and their implications. Furthermore, our study explored the involvement of mi-RNAs and lncRNAs in the overexpression of adhesion molecule genes. Although this analysis provides insights into potential regulatory mechanisms, it is important to note that it is based on computational predictions and would greatly benefit from experimental validation to establish a causal relationship. Lastly, the study suggests the potential utility of the CDC42, TAGLN, and GSN genes as diagnostic or prognostic markers for colorectal cancer. However, to ascertain their clinical applicability, larger-scale validation studies encompassing diverse patient populations are essential. These studies should evaluate the sensitivity, specificity, and predictive value of these genes as biomarkers, while comparing their performance to existing diagnostic and prognostic markers. Addressing these limitations in future research endeavors will facilitate the establishment of the clinical significance and utility of these genes as diagnostic or prognostic markers for colorectal cancer.

## Conclusion

In conclusion, our study identified an association between the increased expression of CDC42, TAGLN, and GSN in high-risk polyps, suggesting a potential role in polyp malignancy. Although CDC42 and TAGLN showed contrasting expression patterns in COAD tissues and polyps, their overexpression in high-risk polyps may still serve as an indicator of malignancy risk. The discrepancy in expression patterns could be attributed to the different roles these genes play in polyp development and cancer progression. Further investigation is required to determine the precise functional mechanisms of these genes in CRC development and to validate their potential as biomarkers for the early detection of high-risk polyps.

Future studies could focus on longitudinal analyses of polyp samples, tracking the progression of low-risk and high-risk polyps over time, as well as examining the potential of CDC42, TAGLN, and GSN as therapeutic targets. Additionally, investigating the interaction between these genes and the immune system could provide valuable insights into their involvement in CRC pathogenesis. Ultimately, a better understanding of the molecular mechanisms underlying polyp malignancy could contribute to the development of more effective prevention and early detection strategies for CRC.

## Data Availability

The experimental data that support the findings of this study are not openly available due to reasons of sensitivity and are available from the corresponding author upon reasonable request. The in silico analyses conducted in this study, as well as the datasets presented, are accessible through online repositories. The names of these repositories, along with the accession numbers, are provided in the article.

## Abbreviations

ABP: Actin-binding protein
AUC: Area under the ROC curve
COAD: Colorectal adenocarcinoma
ECM: Extracellular matrix
EMT: Epithelial-to-mesenchymal transition
FDA: Food and drug adminstration
GAPDH: Glyceraldehyde 3-phosphate dehydrogenase
GO: Gene ontology
GSN: Gelsolin
HRA: High risk adenomas
KEGG: Kyoto encyclopedia of genes and genomes
lncRNA: Long non-coding RNA
LRA: Low-risk adenomas
PPI: Protein–protein interaction
Q-PCR: Quantitative polymerase chain reaction
ROC: Receiver operating characteristic
TAGLN: Transgelin
TCGA: The cancer genome atlas
